# Teratoma Growth Retardation by HDACi Treatment of the Tumor Embryonal Source

**DOI:** 10.3390/cancers12113416

**Published:** 2020-11-18

**Authors:** Jure Krasic, Lucija Skara, Monika Ulamec, Ana Katusic Bojanac, Sanja Dabelic, Floriana Bulic-Jakus, Davor Jezek, Nino Sincic

**Affiliations:** 1 Department of Medical Biology, School of Medicine, University of Zagreb, 10 000 Zagreb, Croatia; jure.krasic@mef.hr (J.K.); lucija.skara@mef.hr (L.S.); ana.katusic@mef.hr (A.K.B.); floriana.bulic@mef.hr (F.B.-J.); 2 Scientific Group for Research on Epigenetic Biomarkers, School of Medicine, University of Zagreb, 10 000 Zagreb, Croatia; monika.ulamec@gmail.com; 3 Centre of Excellence for Reproductive and Regenerative Medicine, School of Medicine, University of Zagreb, 10 000 Zagreb, Croatia; davor.jezek@mef.hr; 4 Ljudevit Jurak Clinical Department of Pathology and Cytology, Sestre Milosrdnice University Hospital Center, 10 000 Zagreb, Croatia; 5 Department of Pathology, School of Medicine, University of Zagreb, 10 000 Zagreb, Croatia; 6 Department of Biochemistry and Molecular Biology, Faculty of Pharmacy and Biochemistry, University of Zagreb, 10 000 Zagreb, Croatia; sanja.dabelic@pharma.unizg.hr; 7 Department of Histology and Embryology, School of Medicine, University of Zagreb, 10 000 Zagreb, Croatia

**Keywords:** HDACi, TGCT, epigenetics, cancer stem cells, valproate, Trichostatin A

## Abstract

**Simple Summary:**

Testicular germ cell tumors are the most common neoplasms in young male populations, with a rising incidence. Among them, teratomas may often be very aggressive and resistant to therapy. Our aim was to investigate the impact of two potential anti-tumor epigenetic drugs (Valproate and Trichostatin A) in a mammalian model of teratoma development from an early trilaminar mouse embryo. Both drugs applied to the embryonic tissue had a significant negative impact on the teratoma growth in a three-dimensional in vitro culture. However, Trichostatin A did not diminish some potentially dangerous features of teratomas in contrast to Valproate. This research is an original contribution to the basic knowledge of the origin and development of teratomas. Such knowledge is necessary for envisioning therapeutic strategies against human testicular tumors.

**Abstract:**

Among testicular germ cell tumors, teratomas may often be very aggressive and therapy-resistant. Our aim was to investigate the impact of histone deacetylase inhibitors (HDACi) on the in vitro growth of experimental mouse teratoma by treating their embryonic source, the embryo-proper, composed only of the three germ layers. The growth of teratomas was measured for seven days, and histopathological analysis, IHC/morphometry quantification, gene enrichment analysis, and qPCR analysis on a selected panel of pluripotency and early differentiation genes followed. For the first time, within teratomas, we histopathologically assessed the undifferentiated component containing cancer stem cell-like cells (CSCLCs) and differentiated components containing numerous lymphocytes. Mitotic indices were higher than apoptotic indices in both components. Both HDACi treatments of the embryos-proper significantly reduced teratoma growth, although this could be related neither to apoptosis nor proliferation. Trichostatin A increased the amount of CSCLCs, and upregulated the mRNA expression of pluripotency/stemness genes as well as differentiation genes, e.g., *T* and *Eomes*. Valproate decreased the amount of CSCLCs, and downregulated the expressions of pluripotency/stemness and differentiation genes. In conclusion, both HDACi treatments diminished the inherent tumorigenic growth potential of the tumor embryonal source, although Trichostatin A did not diminish the potentially dangerous expression of cancer-related genes and the amount of CSCLC.

## 1. Introduction

Testicular tumors are the most common malignancy in men of European ancestry aged between 15 and 44 years [1,2]. In 2018, more than 71,000 cases of testicular cancer were diagnosed, with over one-third being in Europe. Testicular germ cell tumors (TGCT) make up around 95% of all testicular cancer cases and arise from the same precursor, germ cell neoplasia in situ (GCNIS) [3]. TGCTs have a cure rate of over 95% after orchiectomy and cisplatin-based chemotherapy. However, in 15–30% of patients with disseminated disease, relapse and cisplatin resistance can develop, for which there is no effective treatment [1,4,5,6,7]. Overall, around 36 years of life are lost per man dying of TGCT [5]. TGCTs are divided into seminoma and non-seminoma subtypes. They occur in around the same ratio (1:1) [3], and seminomas, which are non-differentiated tumors, exhibit the highest cure rate, while the chemotherapy sensitivity of extraembryonic differentiation varies from very high to very low, suggesting differentiation might be a factor in outcome [8,9]. Teratoma being terminally differentiated is resistant to chemotherapy, and the presence of teratoma is associated with both early and delayed disease-mortality [8].

Unlike prepubertal testicular teratomas, which are not GCNIS-derived, postpubertal teratomas are always malignant and tend to metastasize in about 30% of cases [9,10]. Postpubertal teratoma can occur in prepubertal patients, especially at under three years of age [11]. This rarely appears as a pure form—only in about 5% of all TGCT cases, and more often as a component of mixed TGCT. The diagnosis of teratoma still relies primarily on classical histopathological and immunohistochemical (IHC) methods of detection [1,2,12].

In 1967, Stevens transplanted mouse genital ridges and embryos into mouse testis, and in 1971 Damjanov, Solter, Belicza and Skreb described the effects of the transplantation of 7.5-day-old mouse egg-cylinders under the kidney capsule of syngeneic adult mice. In both series of experiments, from normal embryonal tissue, malignant teratoma developed [13,14]. It was proposed that the ectopic environment, through epigenetic mechanisms, induces a blockage in the differentiation and malignant transformation of the mammalian embryonal cells, which should be more important for the development of such “developmental” tumors than mutations. Additionally, the transplanted rat egg-cylinders produced mostly benign tumors cultivated in vitro, which were used to test the effects of various agents or culture conditions on the growth, differentiation and apoptosis of the resulting experimental teratoma [15,16]. In this research, for the first time, we treated the normal mouse embryonal tissue with epigenetic drugs to investigate the impact of histone acetylation on the source of the teratoma.

It was proposed that the ectopic environment through epigenetic mechanisms induces a blockage in differentiation and malignant transformation of the mammalian embryonal cells, and that this epigenetic regulation is more important for the development of these “developmental” tumors than mutations. Importantly, the model has been used to test the effects of various agents or culture conditions on the growth, differentiation and apoptosis of resulting experimental teratoma in vitro [7,15,16].

Recently, the search for the epigenetic molecular mechanisms involved in developmental tumorigenesis has become a hot scientific focus. Teratomas became of interest to not only cancer biologists, but also to germ and stem cell biologists as well, since they provide insight into the interplay between stemness/pluripotency, differentiation and neoplastic transformation [16,17,18,19].

Chromatin modifications, including histone acetylation, control the switch between active euchromatin and repressed heterochromatin, with the acetylation of histones leading to increased gene expression and euchromatin state [20,21,22]. The acetylation of histones has been linked with the regulation of various biological processes such as differentiation, pluripotency/stemness and self-renewal, but also with a number of diseases, including cancer [23]. Histone acetylation is primarily regulated by histone acetyltransferases (HATs) and histone deacetylases (HDACs). There are 18 HDACs that are divided into classes I, II, III and IV, based on their function and sequence similarity [20,21,24,25]. The use of HDAC inhibitors (HDACi) as therapy for neoplastic diseases is a decades-old idea [23,26] that culminated in the FDA approval of HDACi cancer drugs [21]. HDACi are small molecules that inhibit the action of HDACs and increase the acetylation levels of histones, thereby producing an effect on a whole variety of biological processes, such as apoptosis, autophagy, senescence, growth arrest, differentiation and cell cycle arrest [23,24,27]. In fact, it was shown that in mouse and human TGCT cell lines, HDACi treatment can downregulate stemness genes and promote differentiation [21]. In this research, we treated the normal embryonal tissue (mouse egg-cylinders) with two prominent HDACi, Trichostatin A (TSA) and Valproate (VPA), to investigate the impact of histone acetylation on the source and development of the teratoma.

TSA is a complete class I and II HDACs inhibitor [26,28], which was originally used as an antimycotic [29]. It increases histone acetylation and consequently has a strong impact on oxidative stress, cell cycle arrest, apoptosis [28,30,31], and epithelial–mesenchymal transition [32]. Its anti-tumor effect has been shown in multiple types of cancer, such as, colon, breast, prostate and esophageal squamous cells [28,30,31]. TSA has on its own induced the suppression of angiogenesis and metastasis in cancer cells, as well as the suppression of vasculogenic mimicry in glioblastoma cell lines [29,30,33]. In combination with other therapies it has been documented to reverse resistance to chemotherapy [29,30]. However, the molecular mechanisms by which TSA induces all these effects are not fully understood [29].

VPA is used clinically as an anti-epileptic agent and for the treatment of neurological disorders [15,34,35,36]. It inhibits class I and IIa HDACs, which increases histone acetylation and protein synthesis in cells, preventing their proliferation, and ultimately resulting in tumor cell differentiation [20,34]. This, combined with its effect on cellular reprogramming, senescence and the promotion of apoptosis, led to its use in breast cancer adjunct therapy, and multiple clinical trials for cancer therapy [15,34,35,37]. The anti-tumor effect of VPA has been documented in TGCT cell lines, including choriocarcinoma [38]. Still, the molecular mechanisms of VPA are not fully understood, which is exemplified by numerous side effects continuously being discovered [35,37,39].

The mechanism of HDACi’s effect on TGCT seems to operate via growth reduction and terminal cell differentiation. However, multiple studies report TSA and VPA treatment promoting and restoring pluripotency, and dedifferentiating somatic cells [40,41,42]. VPA and TSA both have effects on mammalian embryo development, with VPA being a known teratogenic substance [43,44], while TSA was reported to increase the survival rate of embryos fertilized in vitro [45,46]. Conversely, both VPA and TSA have been described as having an anti-tumor effect on TGCT cell lines [47,48].

In this study we aimed to investigate the impact of two prominent HDACi, TSA and VPA, on the potential of the embryo-proper, consisting only of the three germ layers (ectoderm, mesoderm and endoderm), to develop into teratoma. Indeed, a short treatment of the embryo-proper with HDACi before cultivation diminished the subsequent in vitro growth of teratomas.

## 2. Results

### 2.1. Histopathology of Teratomas

**Figure 1:**
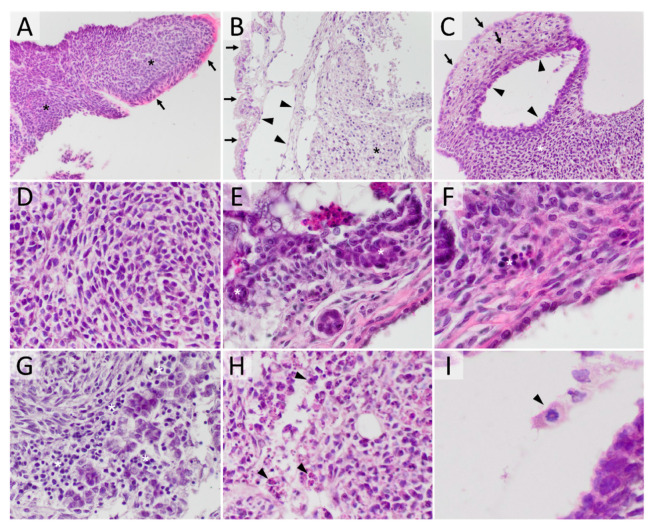
Experimental mouse teratoma morphology. (**A**) teratoma consisting mostly of undifferentiated tissue (asterisk—cords and sheets of mainly monomorphic cells with high mitotic activity and without any visible forming structure), while on the surface clearly differentiated tissue is present (arrow—morphological cylindrical epithelial cells with eosinophilic cytoplasm and pseudostratified epithelial cells) (HE, 200×); (**B**) teratoma composed of differentiated tissue (arrow—surface epithelial cells, arrowhead—cystic structure lined by thin flattened epithelia), while the inside is mostly made up of UTC (asterisk) (HE, 200×); (**C**) the teratoma composed of all three germ cell-derived lines, mesodermal (asterisk), endodermal (arrowhead) and ectodermal (arrow) (HE, 200×); (**D**) high magnification of UTCs (HE, 400×); (**E**) high magnification of CSCLC (asterisk) (HE, 400×); (**F**) high magnification view of hematopoiesis (asterisk) (HE, 400×); (**G**) high magnification of lymphocytes (asterisk) (HE, 400×); (**H**) high magnification of cells in the process of apoptosis (arrowhead) (HE, 400×); (**I**) high magnification of a cell in the process of mitosis (arrowhead) (HE, 600×).

The professional pathologists’ criteria for identifying tissue and cell types were used in the comprehensive analysis of teratomas. Teratomas contained undifferentiated teratoma cells (UTC)—cells displaying no signs of atypia or malignancy while showing no signs of differentiation. Moreover, they contained cancer stem cell-like cells (CSCLCs)—large pleomorphic cells displaying malignant nuclear criteria, anisokaryotic hyperchromatic nuclei, a high nuclear cytoplasmic ratio and a high percentage of mitotic activity [49]. Teratomas also contained tissue displaying clearly noticeable structures or cell types. The different types of cells and tissues found in teratomas are presented in Figure 1.

The control teratomas consisted of both undifferentiated tissue and tissue in the process of differentiation in the ratio 1:1. A small percentage of necrosis was detected in some teratoma, which made up 1% of total teratoma tissue. In most teratoma, the presence of inflammatory cells, leukocytes, was found. These inflammatory cells made up around 11% of the total teratoma tissue. Cells in the process of apoptosis and mitosis were also detected in both tissue components, with the apoptotic index of 0.6 and mitotic index of 5.5 in differentiated tissue, and in undifferentiated tissue the apoptotic index of 1.7 and a much higher mitotic index of 21.5. The differentiating tissue originating from the ectoderm constituted 42%, mesodermal tissue 13%, and endodermal tissue 1% of the total teratoma tissue. The undifferentiated teratoma tissue was mostly comprised of UTC with an admixture of morphologically different cells, which fit the description of cancer stem cells. Since this is the first instance of observing and describing these cells in our teratoma in vitro model, we designated them CSCLCs.

**Figure 2:**
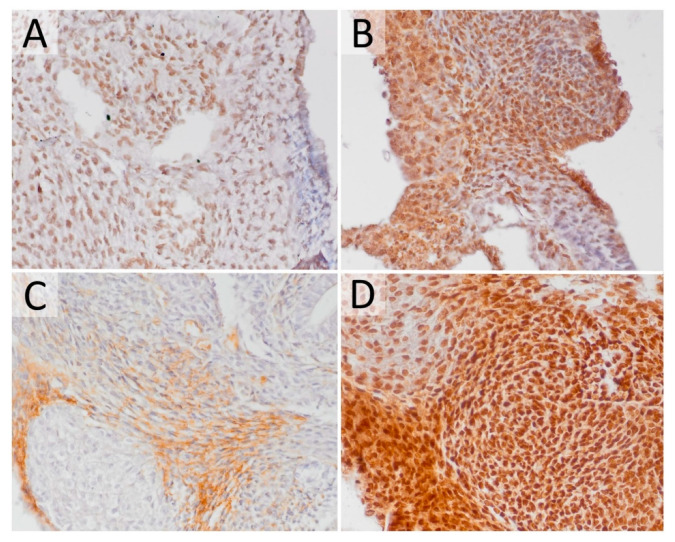
Experimental mouse teratoma gene expression on the protein level. (**A**) Most cells exhibit a positive signal for POU5F1 (IHC, 400×); (**B**) most cells exhibit a positive signal for NANOG (IHC, 400×); (**C**) cytoplasmic positivity of p53 in differentiated cells (IHC, 400×); (**D**) most cells exhibit a positive signal for 8-OHdG (IHC, 400×).

The assessment of molecular differences between the differentiated and undifferentiated components and the potential difference between the UTC and CSCLCs was performed by IHC analysis. We have selected *Pou5f1* (*Oct4*) and *Nanog* gene expression as stemness/pluripotency markers, and the tumor suppressor gene *p53* and 8-OHdG (8-hydroxy-2’-deoxyguanosine) as prominent markers of oxidative stress and DNA damage (Figure 2). *Pou5f1* and *Nanog* have shown uniform expression across all teratoma cells, both differentiated and undifferentiated. The expression of *p53* was only detected in clearly differentiated tissue (e.g., fibroblasts) and was localized in the cytosol. 8-OHdG was expressed uniformly across both differentiated and undifferentiated tissue. UTC and CSCLCs have both shown identical expressions of the investigated markers.

### 2.2. The Effect of HDAC Inhibition on Teratoma Growth

**Figure 3:**
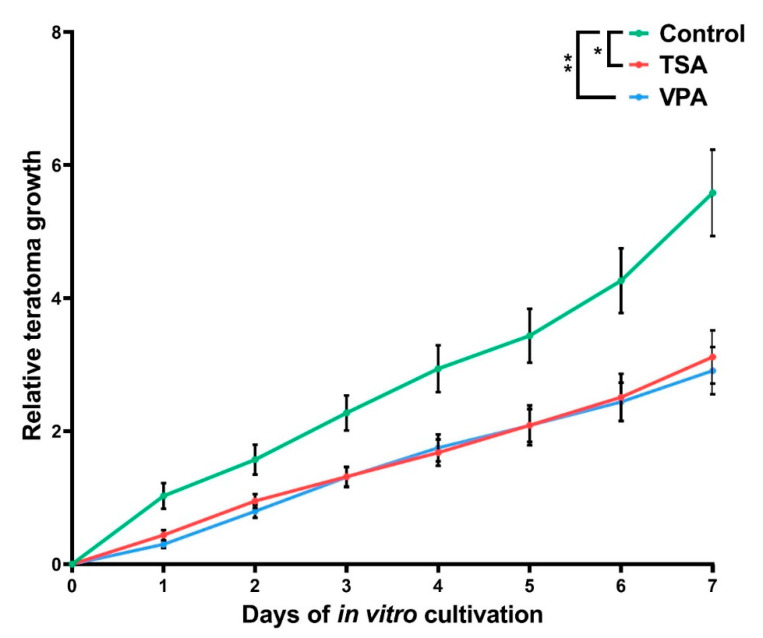
Teratoma growth during in vitro cultivation. The measured size of each teratoma was normalized to the measured size at day 0 of sample plating. Values represented are means with 95% CI. Asterisks were used to depict statistical significance, * *p* < 0.05, ** *p* < 0.01.

The cultured teratomas have shown a relative increase in size of 5.6 times in the control group, 3.1 in the TSA group and 2.9 in the VPA group (Figure 3). Compared to the controls, HDACi-treated groups had growth retardation of almost 50% (44% TSA, 48% VPA). Overall, the HDACi treatments have exhibited the functionally identical and statistically significant growth retardation of in vitro-cultivated teratomas.

### 2.3. The Effect of HDACi on Histone Acetylation in Teratomas

**Figure 4:**
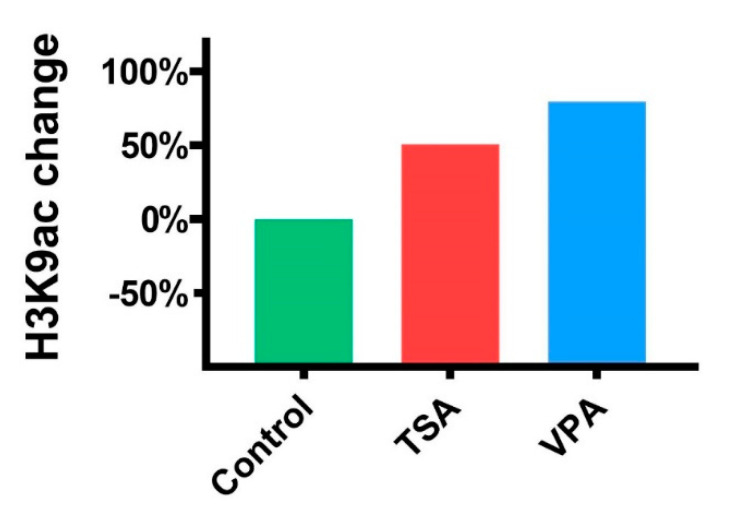
Acetylated histone H3K9 expression change compared to the control group.

To confirm the activity of HDACi treatment on histone acetylation in treated teratomas, we have used Western blotting to semi-quantify histone H3K9 acetylation. Both HDACi treatments increased histone acetylation compared to the control group, with TSA treatment increasing acetylation by 50% and VPA by 79% (Figure 4).

### 2.4. HDACi Effect on Teratoma Differentiation

**Figure 5:**
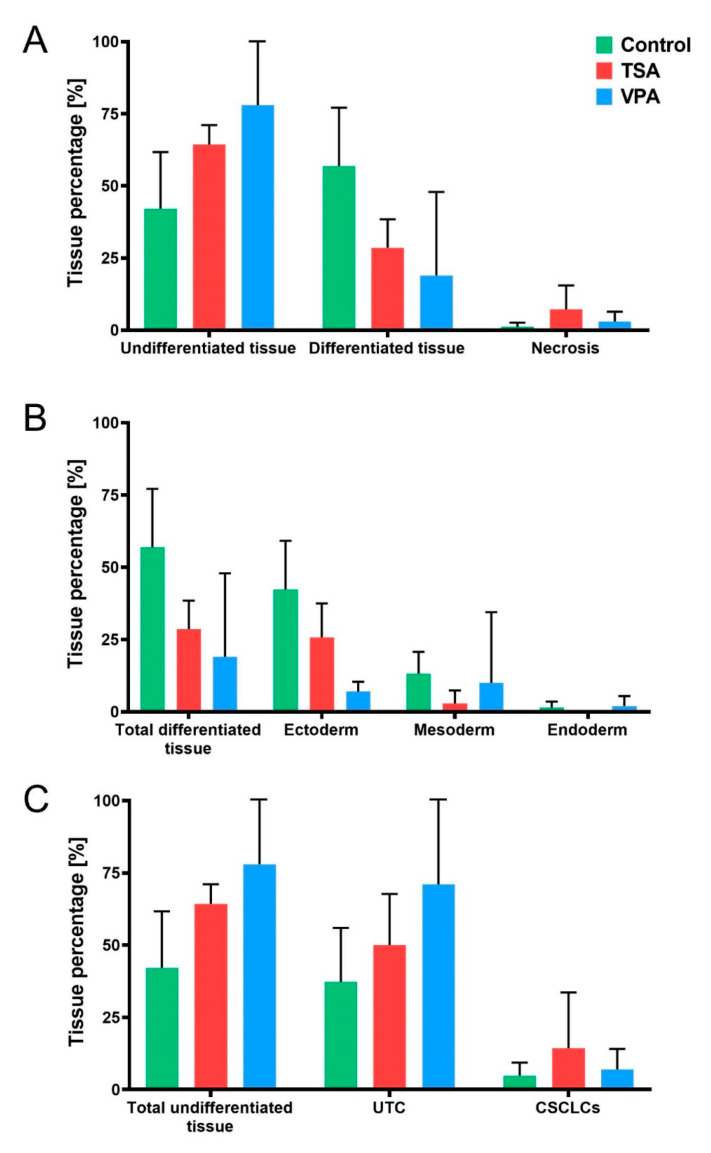
Teratoma tissue distribution. (**A**) Teratoma tissue components across treatment groups, divided into undifferentiated tissue, differentiated tissue and necrotic tissue. (**B**) Tissue differentiation according to germ layer of origin, with percentage of differentiated tissue depicted as belonging to ectodermal, mesodermal or endodermal tissue across treated groups. (**C**) Stratification of undifferentiated teratoma components into undifferentiated teratoma cells (UTC) and cancer stem cell-like cells (CSCLCs). Values represented are means with 95% CI.

With the TSA and VPA treatments being effective both in increasing acetylation levels and in diminishing teratoma growth, an assessment of their impact on teratoma differentiation was performed by HE morphometric analysis (Figure 5). TSA-treated teratoma had 29% differentiated tissue, those VPA-treated had 19%, while differentiated tissue made up 57% of the control teratomas. Undifferentiated tissue made up 64% in the TSA treatment, 78% in the VPA treatment and 42% of the control teratomas. The remainder was necrotic tissue, which made up 1% of the control, and 7% of the TSA-treated and 3% of the VPA-treated teratomas (Figure 5A).

After assessing overall teratoma differentiation, we performed the stratification of differentiated tissue according to germ layer of origin. HE morphometric analysis further revealed that ectodermal tissue made up 26% of TSA-treated teratoma, 7% of VPA-treated and 42% of control teratomas (Figure 5B). Mesodermal tissue made up 3% of TSA-treated teratomas, 10% of VPA-treated and 13% of control teratomas. Endodermal tissue was absent in TSA-treated teratomas, while in the VPA group it made up 2% of the tissue and it constituted 1.4% of the control group.

An “anti-inflammatory-like” effect of both HDACi treatments was noticed, with lymphocytes making up 3% of both TSA- and VPA-treated teratoma, compared to 10% in controls.

A further stratification of the effects of the HDACi treatments on undifferentiated tissue, that is to say, the ratios of UTC and CSCLCs, has been analyzed. VPA-treated teratomas have the UTC/CSCLCs ratio of 10:1 in favor of UTC, as compared to the 7:1 ratio of control teratomas. TSA-treated teratoma have the ratio of 4:1, with the highest amount of CSCLCs (Figure 5C).

### 2.5. Proliferation and Apoptosis

**Figure 6:**
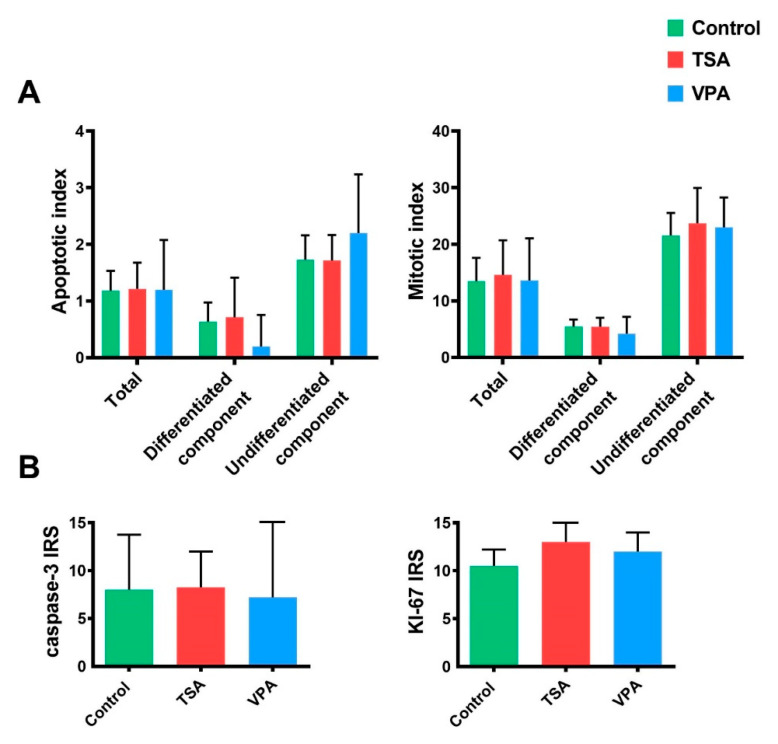
Analysis of apoptosis and proliferation. (**A**) Apoptotic and mitotic indices have been measured in in vitro-cultured teratoma HE slides as total number of apoptotic or mitotic cells in differentiated and undifferentiated teratoma tissue. Values represented are means with 95% CI. (**B**) IHC analysis of apoptosis and proliferation in in vitro-treated teratoma. Proliferation has been analyzed based on *Ki-67* protein expression, while apoptosis by caspase-3 expression. Values represented are means with 95% CI.

Morphological and molecular methods were both used to assess the effect of HDACi treatment on teratoma development so as to determine if growth retardation could be the result of either increased apoptosis or reduced proliferation. On the morphological level, we have morphometrically analyzed the apoptotic and mitotic indices of in vitro-cultivated teratoma on HE slides. No significant change in apoptosis or mitosis in the HDACi-treated groups was found (Figure 6A).

On the molecular level, we have analyzed the gene expression of *Ki-67*, a known cellular marker of proliferation, and the expression of active caspase-3, a central executor of cellular apoptosis on the protein level, using IHC (Figure 6B). As in the previous analysis on HE slides, no differences between the HDACi-treated groups and the control group were found.

### 2.6. Oxidative Stress Levels

**Figure 7:**
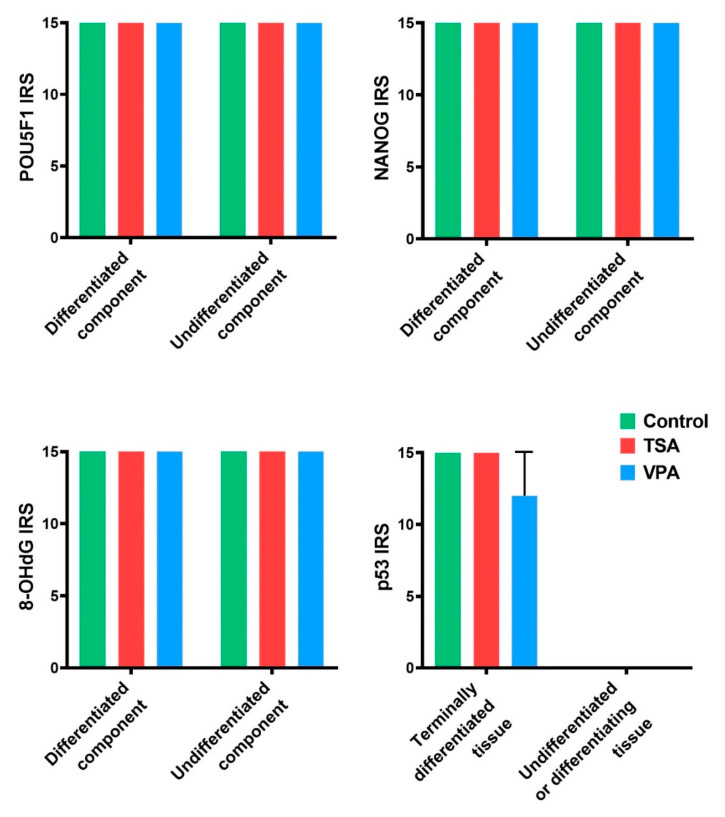
IHC analysis of in vitro treated teratomas, separated by tissue component. Values represented are means with 95% CI.

DNA ROS-induced damage has been analyzed via morphometric analysis of IHC teratoma slides for 8-OHdG expression. The treated teratomas exhibited the same expression of the oxidative stress marker (Figure 7).

### 2.7. Gene Expression Analysis

**Figure 8:**
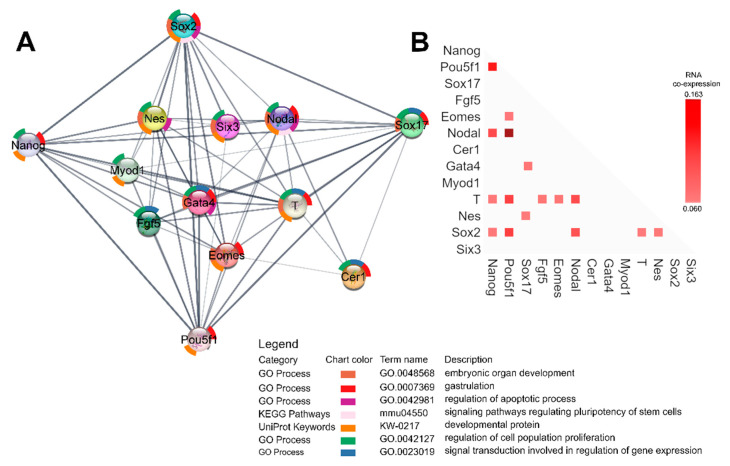
(**A**) STRING functional gene analysis using Cytoscape. Each node represents a protein product of an investigated gene. Lines denote protein–protein interactions, with line thickness being indicative of evidence strength for a predicted interaction. (**B**) RNA co-expression, by STRING v11. Each node represents evidence of intersecting genes’ RNA co-expression. Scores are represented with lighter hues of red for weaker and darker hues for stronger.

To confirm the biological roles of the genes involved in this study we have performed functional gene enrichment analysis (Figure 8A). With 13 nodes, the analyzed network has 57 edges, while the expected number of edges was 3, meaning a significant PPI enrichment *p*-value. Enrichment like this indicates that the investigated proteins are likely connected in a biological group. The functional enrichment results show the involvement of the investigated genes in biological processes with FDR < 0.05, such as gastrulation, signal transduction involved in regulation of gene expression, embryonic organ development, regulation of apoptotic processes and regulation of cell population proliferation. The investigated genes also had a statistically significant FDR labeling them as developmental proteins by UniProt analysis, and a KEGG analysis identifying members of the signaling pathway regulating the pluripotency of stem cells. This confirms the strong connection of the genes involved in proliferation and apoptosis, as well as the early trilaminar differentiation and stemness pathways, with often the same genes being involved in both. Indeed, RNA co-expression analysis has shown that the stemness and differentiation genes are often co-expressed (Figure 8B). Indeed, the highest RNA co-expression score is between *Pou5f1* and *Nodal*, followed by *Nanog* and *Pou5f1*, *Pou5f1* and *Sox2*, and *Pou5f1* and *T.* This interconnectivity of stemness-differentiation genes well describes the interdependence of the stemness and differentiation processes.

**Figure 9:**
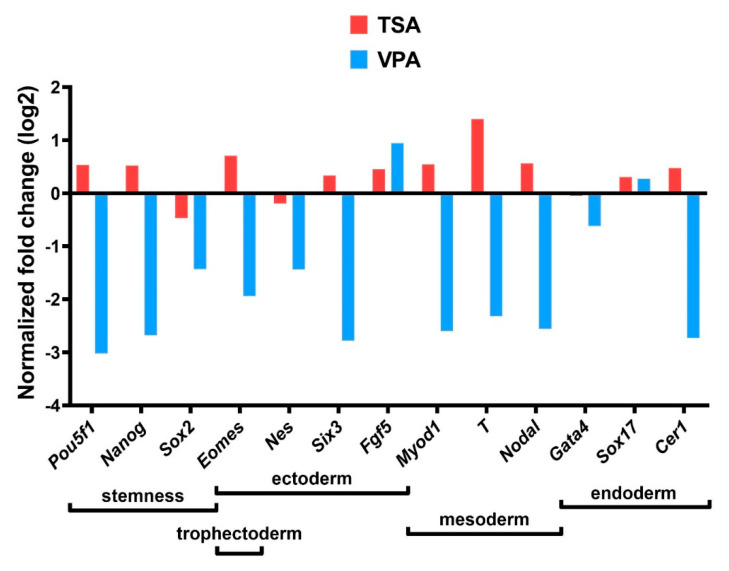
HDACi treatment effect on gene expression. Gene expression on mRNA level analyzed by qPCR, all values normalized to the control group and log-transformed.

Gene expression analysis of mRNA levels (Figure 9) has shown that TSA treatment downregulated *Sox2*, *Nes* and *Gata4* compared to the control group, while *Pou5f1*, *Nanog*, *Eomes*, *Six3*, *Fgf5*, *Myod1*, *T*, *Nodal*, *Sox17* and *Cer1* were upregulated. VPA treatment, on the other hand, upregulated *Sox17* and *Fgf5* compared to the control group, while all other genes were downregulated.

Gene expression on the protein level has been analyzed by a morphometric analysis of IHC teratoma slides. The *Pou5f1*, *Nanog* and *p53* gene expressions in the treated teratoma showed similar expressions to those in the control teratomas. *Pou5f1* and *Nanog* were expressed in the whole teratoma, and *p53* expression was only present in the most differentiated tissue, which was epithelium for TSA treatment and mesenchyme for VPA treatment (Figure 7).

## 3. Discussion

### 3.1. Histopathological and Molecular Analysis of the Experimental Teratoma In Vitro System

A rodent teratoma in vitro system has been used to assess the impacts of various biologically active substances and therapeutics on the developmental processes important for teratoma tumorigenesis [15,16]. As studies show that in humans, both immature and mature teratoma may possess malignant potential [50], we performed for the first time a comprehensive histopathological analysis and an analysis of early developmental markers during the active growth phase of the mouse teratoma system in vitro. The advantage of this system is a natural three-dimensionality that is absent from the various two-dimensional in vitro cell cultures often used in cancer research [15]. The cultivated teratomas exhibited a ratio of undifferentiated to differentiated tissue components of 1:1, the apoptotic/mitotic ratio being significantly in the favor of mitosis, while the high protein expression of the pluripotency/stemness markers *Pou5f1*, *Nanog* and the proliferation marker *Ki-67* was assessed in both components. Therefore, the cultivation of experimental mouse teratomas for 7 days yielded proliferating teratomas with cells that retained pluripotency typical for ESCs derived from the inner cell mass, and GCNIS precursors of TGCTs that are thought to be germ cells of impaired differentiation [15,16,51]. The high concentration of the ROS marker 8-OHdG found in teratomas could be due to the very nature of in vitro cultivation, as it may have genotoxic [52] or even epigenetically regulated developmental consequences [53], which should be investigated further.

We have detected the presence of lymphocytes in cultivated teratomas that have differentiated from the embryo-proper, known to be the source of all cells and tissues of an organism, which in our case is devoid of any extra-embryonal parts. To our current knowledge, we are the first to describe endogenous immune cell differentiation in experimental teratoma in vitro. The immune cells in our system probably originate from the detected sites of ongoing hematopoiesis, derived from the embryonal mesoderm. It must be noted that the embryonal mesoderm may develop hematopoietic cells [54], and their origin may be something other than the classical extra-embryonal mesoderm of the yolk sac. The presence of hematopoietic sites has been documented also in teratomas obtained in vivo from iPSCs, and this particular system was proposed for the generation of HSCs for clinical purposes [55]. While immune cell infiltration has generally been associated with seminoma histology and favorable prognosis [1], a deeper investigation of non-seminoma immune cell infiltration has shown significant leukocyte presence [56,57]. According to our results, it is possible some of the lymphocytes detected in tumors in vivo may result from the process of differentiation, and not only from infiltration from extraneous sources.

Importantly, CSCLCs have also been observed for the first time in cultivated teratomas, these being a small population of cells within tumors that hold stemness properties that sustain cancer progression [58]. They generally arise from “normal” stem cells, such as ES or iPSC, cells grafted in vivo or the cells of the trilaminar embryo, or may even arise as a result of a blockage in the differentiation or dedifferentiation of somatic cells due to genetic, epigenetic and environmental factors [15,59].

### 3.2. Teratoma Growth In Vitro

Both HDACi treatments produced very similar overall growth retardation effects, consistent with previous reports that may be especially important for therapeutic strategies [15,48]. Cell cycle arrest due to TSA and VPA treatment in the G1, G2 and even S phases is well documented [60,61], and cell cycle arrest-inducing substances are being increasingly investigated for their anti-tumor and pro-apoptotic properties [62,63]. The marked reduction in the lymphocyte presence in both treatments points to a slowing down of teratoma development, since studies have previously shown that a reduction in pro-inflammatory markers and an inhibition of inflammation reduces in vivo tumor size and promotes apoptosis and autophagy [64]. Additional molecular investigations would shed light on the exact lymphocyte role in our case.

While HDACi treatment was reported to increase apoptosis in the rat experimental teratomas [15], we have found no such increase. This difference may be dose-dependent, since the rat embryos/teratomas were treated with higher doses of VPA, and were treated multiple times during a period of 14 days, while in the present experiment they were treated just before in vitro plating. A stronger effect of high HDACi dose as related to higher acetylation levels was reported before, even with descriptions of certain cell types, namely CSCs, requiring higher doses for the same desired effect [33,42,48]. Therefore, our results show the impact of HDACi on the trilaminar origin of the teratoma itself, rather than the already developing tumor. Some of the results of our molecular analyses also deviate from expectations related to apoptosis. Reactive oxygen species (ROS) accumulation in the cells is one of the mechanisms by which HDACi treatments are known to activate apoptosis [61,65]. We have also observed p53 cytoplasmic expression in differentiated teratoma components, and p53 may induce mitochondrial ROS production in order to activate apoptosis, usually due to genotoxic stress [66]. Furthermore, our gene enrichment analysis has shown that genes involved in the regulation of apoptosis, *Gata4*, *Sox2* and *Nes*, were downregulated in both HDACi treatments. *Gata4*, *Sox2* and *Nes* are anti-apoptotic genes, and their downregulation should promote apoptosis, which we did not find in the treated teratomas. In the case of already malignant cells, such as F9 teratocarcinoma cells, transformed hematopoietic progenitor cells and leukemic blasts from acute myeloid leukemia patients, VPA is known to induce differentiation and apoptosis in vitro, and reduce the tumor growth of breast cancer cells transplanted into rats in vivo [67]. Ultimately, the lack of observed difference in apoptosis between treated and untreated groups may be due to the specificity of the experimental mouse system, i.e., we treated normal embryonal tissue and not malignant cells. Interesting to note is that even various types of pluripotent stem cells can induce teratomas with different efficiencies and speeds, regardless of the in vivo microenvironment [68].

It is well known that TSA and VPA cause the differentiation of pluripotent cells such as EC cells [48], but on the contrary, may dedifferentiate somatic cells into a pluripotent state, suggesting that both states are regulated by the same mechanism [40,69]. There are reports of TSA and VPA treatment increasing embryonal stem cell self-renewal capacity [40,42], and restoring the pluripotency of amniotic fluid stem cells [41], with increased histone acetylation interfering with differentiation [40,42]. Our results show that in both treated groups, the ratio of differentiated and undifferentiated tissue components shifted in the favor of undifferentiated tissue. However, the ratio of UTC to CSCLCs did not increase equally. VPA treatment increases the ratio in favor of UTC, while TSA increases this ratio more in favor of CSCLCs. Therefore, TSA treatment could have produced an additional dedifferentiating effect so as to produce cells that are more susceptible to malignant transformation [68], resulting in an increased ratio of CSCLCs. As for differentiation, the VPA-treated group had all three germ layer tissue derivatives present, while in the TSA-treated group no endodermal tissue was found. This could very well suggest that the primary mechanism of VPA action is in inducing cell cycle arrest, while TSA, along with inducing cell cycle arrest, might induce tissue dedifferentiation as well. Our results and these reports point to the sensitivity and specificity of HDACi treatment response, with histone acetylation level, gene expression profile, differentiation stage and microenvironment all playing a role in determining the response direction.

### 3.3. Gene Expression

Studies have shown that most genes in the VPA-treated ES cells show no difference in expression, meaning global hyperacetylation has little effect on most genes’ expressions or differentiation states, while among the remaining affected genes, both upregulation and downregulation were found [40,69]. This implies that a small subset of genes being sensitive to HDACi treatment may cause a wide array of phenotypical and developmental changes. The genes downregulated by HDACi treatment are often the pluripotency genes *Pou5f1*, *Sox2* and *Nanog* [48,70], which studies reported to exhibit the same effect in TSA and VPA treatments. Differentiated cells had their *Pou5f1*, *Sox2* and *Nanog* expressions restored with increased acetylation [37]. HDACi treatment has produced similar gene expression responses in TGCT cell lines, ES cells and mouse embryos in vivo and in vitro [69,71]. The results of the gene expression analysis that we performed on mRNA level reveal a stark difference in effect between TSA and VPA treatment, with VPA downregulating and TSA upregulating the majority of investigated genes. However, at the protein level there was no difference in *Pou5f1* and *Nanog* expression in teratoma, pointing to the possible mechanism of cell cycle arrest that diminished teratoma growth, rather than the loss of pluripotency within the tissue. This difference in effect on gene expression could be due to the difference in HDACi IIb regulation between VPA and TSA, or the before-mentioned dose-dependent effect [42], with VPA having the greater effect on acetylation levels, or even due to a yet unknown mechanism. The detected discrepancy between mRNA and protein data could also be explained by the fact that embryo cells are less competent in protein translation due to a lack of ribosomes in comparison to the present mRNA quantity [72,73]. Indeed, dissonances between proteome and transcriptome are often reported in embryo and embryo-related models [74]. Congruently, in this study, the detected phenomenon of the RNA expression and protein expression assessments giving different results can be potentially explained by the HDACi-induced change in mRNA level still overwhelming the translation machinery present in the 7-day-old embryo-derived teratomas, or by other mechanisms described to disturb the linearity of the transcription–translation axis.

Worth mentioning is the upregulation of *Fgf5* and *Sox17* in the teratomas originating from both TSA and VPA treatments. The increased *Fgf5* mRNA expression in human melanoma cells in vitro increased clonogenicity and invasion without increasing growth [75], while the *Sox17* in seminoma and primordial germ cells is not a marker of endodermal initiation but supports latent pluripotency [76]. TSA-treated teratomas have shown the highest increase of expression in the *T* gene, which has been associated with worse prognosis in multiple cancer types, including TGCT [77]. This, alongside the increased ratio of CSCLCs, suggests that TSA treatment might have unwanted effects that have to be investigated further. The VPA-treated teratoma, in contrast, seem to be quietened altogether.

## 4. Materials and Methods

### 4.1. Ethical Statement

All animal procedures were conducted according to the Directive 2010/63/EU and those of Croatian Law on the protection of experimental animals. They were approved by the Ethical Committee of the School of Medicine, University of Zagreb, Croatia (380-59-10106-15-168/90, 23 April 2015).

### 4.2. Animals

C3H inbred mice between 2.5 and 3.5 months old, weighing about 25 g, were obtained from the registered animal facility for laboratory rodents at the University of Zagreb School of Medicine, Department of Medical Biology.

The mice were kept in conventional cages, provided with standard diet and bedding with GLP certificate. Food and water were given *ad libitum*. Environmental conditions were constantly monitored; room temperature at 20–24 °C, relative humidity 40–70%, day/night cycle 12/12 h, noise level under 60 dB.

### 4.3. Isolation of the Embryo-Proper

**Figure 10:**
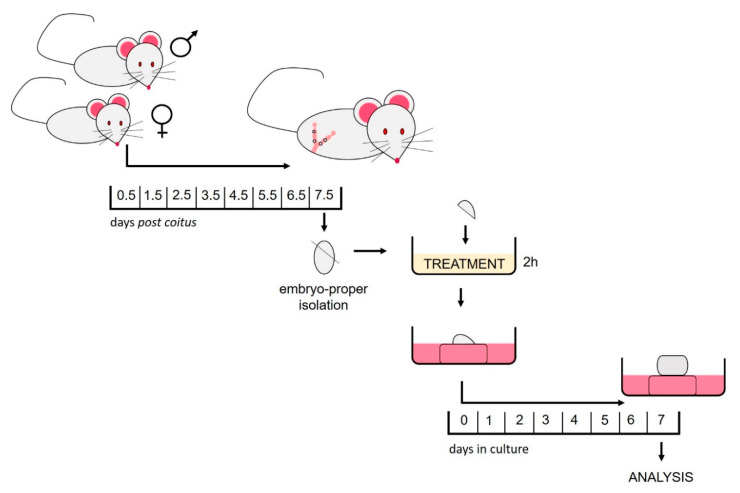
Depiction of the experimental design.

Males were caged alongside females overnight. In the morning, the females were assessed for the presence of a vaginal plug, which was declared as 0.5 day postcoitus (dpc). At 7.5 dpc, the females were euthanized using cervical dislocation. The egg-cylinders were removed from the uteri under a dissecting microscope with a watchmaker’s forceps. After the removal of Reichert’s membranes and ectoplacental cones, the extraembryonic portion was cut at the level of the amnion to isolate the gastrulating embryo-proper which consists of the egg-cylinder composed only of the three germ layers, the ectoderm, mesoderm and endoderm (Figure 10).

### 4.4. HDACi Treatment

After isolation, the embryos-proper (egg-cylinders) were immersed for two hours at room temperature in 250 μL of HDACi enriched medium. Controls were immersed in 250 μL of the non-enriched medium. The medium was made in a 1:1 ratio by combining Eagle’s minimum essential medium (MEM) with Hank’s balanced salt solution and rat serum. HDACi were added to the experimental medium to the final concentration of 1 mM for Valproate (Sigma-Aldrich, St. Louis, MO, USA) [78] and 33 nM for Trichostatin A (Sigma-Aldrich) [79].

### 4.5. In Vitro Culture

As previously described, treated egg-cylinders were cultivated at the air–liquid interface. In short, they were placed on a lens paper supported by a stainless-steel grid in a 60 × 15 mm center well organ culture dish (BD FalconTM, Oxford, UK) with enough medium in the well to wet the lens paper [15]. Cultivation medium consisting of 1:1 Eagle’s MEM with Hanks balanced salt solution and rat serum was used. The plating day was considered as day 0 of culture. The teratomas were cultivated for 7 days at 37 °C in 5% CO_2_ and 95% humidified air, with the cultivation medium being changed every 48 h. On the last day of culturing, teratomas were scraped from the lens paper and either stored separately for histopathological analyses or pooled according to the treatment group and stored at −80 °C.

Teratoma in vitro growth was measured non-invasively every day from day 0 of cultivation. Using an ocular micrometer the major and minor diameters were measured and were used in ellipse area calculation (A = π * major diameter * minor diameter/4) [15]. All values were normalized to the initial teratoma size at plating. GraphPad Prism6 software (Kruskal–Wallis test with Dunn’s multiple comparison test) was used to analyze the endpoint growth of 25 control, 18 TSA treated and 22 VPA treated teratomas.

### 4.6. Morphometric Analysis, Hematoxylin and Eosin (HE) and Immunohistochemistry (IHC)

In vitro-cultivated teratomas were fixed for 24 h in mild St. Marie solution (1% glacial acetic acid in 96% ethanol), dehydrated, and embedded in paraffin. Serial sections (4 μm) of teratomas were used for HE or IHC analysis.

For the histological analysis of differentiation as well as apoptotic and mitotic indexes, HE-stained sections were used as previously described [80]. Apoptotic and mitotic indices were calculated as the number of cells in apoptosis (cells with condensed chromatin, cell shrinkage, fragmented nucleus and eosinophilic cytoplasm) or mitosis (cells of typical morphology with nuclei showing condensed chromatin and jagged edges) per one ocular field at 400× magnification of the teratoma’s differentiated and undifferentiated components. The whole surface area was analyzed in low- (40×) and high (400×)-powered fields and the ocular field with the highest number of apoptotic or mitotic cells was always selected [81]. Teratoma features were further quantified by calculating the percentages of undifferentiated, differentiated and necrotic tissue, also with regards to subtype. Statistical analysis of tissue components was performed by GraphPad Prism6 software (Kruskal–Wallis with Dunn’s multiple comparisons test) on 11 control, 7 TSA-treated and 5 VPA treated-teratomas.

For IHC analysis the prepared teratoma microscopic slides were deparaffinized, cleared in xylene and hydrated to TBS in graded alcohol solutions. Steam antigen retrieval was performed after which sections were incubated overnight at 4 °C with primary antibody diluted in 1% BSA/TBS/0.05% Tween-20 (Table S1). Slides were then rinsed in TBS, incubated with 3% H_2_O_2_ to block endogenous peroxidase, rinsed in TBS and incubated with secondary antibody (Table S1). The signal was visualized by DAB chromogen (Dako REAL, K5007, Agilent Technologies, Santa Clara, CA, USA). Slides were counterstained with hematoxylin, embedded, and analyzed under an Olympus Bx53 microscope. Appropriate positive and negative controls were used as quality controls.

Morphometric analyses of IHC stained slides were performed by the pathologist. Staining amount was scored as 0 (no signal), 1 (1–10% positive cells), 2 (11–25% positive cells), 3 (26–50% positive cells), 4 (51–75% positive cells) or 5 (≥76% positive cells). The intensity of staining was scored from 0 to 3 (none, low, medium or high). The semi-quantification of protein expression was presented by immunoreactivity score (IRS) which was calculated by multiplying staining amount score and intensity of staining score, creating a range of 0–15. Statistical analysis of protein expression was performed by GraphPad Prism6 software (Kruskal–Wallis with Dunn’s multiple comparisons test) on six control, TSA-treated and VPA-treated teratomas for Ki-67 analysis, and five control, five TSA-treated and four VPA-treated teratomas for Caspase-3 analysis. Eight control, three TSA-treated and two VPA-treated teratomas were used for the remaining IHC analyses.

### 4.7. SDS-PAGE and Western Blot

Between 35 and 40 samples per group were pooled in 100 μL of RIPA (Tris/HCl pH 8.0 50 mM, NaCl 150 mM, SDS 0.1%, Na deoxycholate 0.5%, Triton X-100 1%, 1% 0.5 M EDTA, and 4% complete EDTA-free protease inhibitor (COEDTAF-RO Roche, Sigma Aldrich, St. Louis, MO, USA) due to the small amount of protein per individual teratoma sample. Pooled teratomas were homogenized by a bead-based homogenizer (Bertin) for 2 min at 5000 rpm. A bicinchoninic acid assay (BCA; Sigma, BCA1) was used to determine the protein concentration of pooled samples using a Uvikon-860 spectrophotometer (Kontron Instruments, Zurich, Switzerland).

SDS electrophoresis and Western blot were performed as previously described [15], following the recommended manufacturers guidelines and buffer formulations (Bio-Rad, Bulletin 6376). After blotting, the membrane was cut along the 20 kDa marker and incubated with the primary antibody. Antibody concentrations are listed in Table S1. The membrane was visualized using the Immobilion Western (Merck-Milipore, Darmstadt, Germany) chemiluminescent on the ChemiDoc XRS + (Bio-Rad, Hercules, CA, USA) and then analyzed in Image Lab 6.0.1 (Bio-Rad, Hercules, CA, USA). After that, the visualization membrane was stripped according to Bio-Rad’s protocol (Bio-Rad, Bulletin 6376, Hercules, CA, USA) and reprobed. Results were first corrected to loading control values, after which they were normalized to the value of the control group.

### 4.8. Functional Enrichment Analysis and RNA Coexpression Analysis

To identify functional interactions between selected genes, their co-expression, their gene enrichment and their functional analysis, the Search Tool for Retrieval of Interacting Gene/Proteins database (STRING, version 11.0., https://string-db.org/) and Cytoscape 3.8.0 (https://cytoscape.org/) were used [82,83]. The STRING database connects genes based on predicted interactions of their protein product, including direct (physical) and indirect (functional) associations. The interactions are determined based on genomic context, experimental data, co-expression, and previous knowledge. For the functional enrichment analysis, results with FDA < 0.05 were considered significant.

### 4.9. RNA Isolation and cDNA Reverse Transcription

Between 25 and 35 samples per group were pooled in 1 mL of Trizol (TaKaRa) due to the small amount of RNA per individual teratoma sample. Later, 200 µL of chloroform was added, samples were shaken for 15 s, incubated at room temperature for 5 min, and then centrifuged for 15 min at 12,000× *g* and 4 °C. The top aqueous layer was transferred to a new 1.5 mL tube and an equal volume of ice-cold isopropanol was added. Samples were incubated at room temperature for 10 min and then the RNA was precipitated by centrifugation for 15 min at 12,000× *g* and 4 °C. The RNA pellet was washed by vortexing with 1 mL of 75% ethanol, and then centrifuging the sample for 10 min at 7500× *g* and 4 °C. The pellet was air-dried and resuspended in 30 µL of RNA-free water.

RNA concentration was measured by NanoDrop 2000 spectrophotometer (Thermo Scientific, Waltham, MA, USA), after which the concentration of the samples was adjusted to 200 ng/µL with RNA-free water, and stored at −80 °C.

Per sample, 1.2 μg of RNA was reverse transcribed with the AffinityScript qPCR cDNA Synthesis kit (Agilent Technologies, Santa Clara, CA, USA) according to the manufacturer’s instructions. The samples’ concentrations were then adjusted to 30 ng/µL cDNA with RNA-free water and stored at −20 °C until further use.

### 4.10. Gene Expression Analysis

A panel of stemness genes (*Pou5f1*, *Nanog* and *Sox2*) as well as genes marking the differentiation of the germ layers (ectoderm—*Nes*, *Six3*, *Fgf5*; mesoderm—*Myod1*, *T*; endoderm—*Gata4*, *Sox17*, *Cer1;* mesendoderm—*Nodal*, *Eomes*; trophectoderm—*Eomes*) was constructed [84,85,86,87,88,89,90,91]. The genes were selected on the basis of the strength of evidence for them as markers. Additionally, the selected genes had to have the same role in human and mouse tissue [92,93].

Real-time PCR analysis was done using 60 ng cDNA per reaction on the ABI PRISM 7300 Sequence Detection System (Applied Biosystems, Foster City, CA, USA) using the Brilliant II SYBR Green QPCR Master Mix (Agilent Technologies), all according to the manufacturer’s instructions, for 40 cycles of 30 s denaturation at 95 °C and 60 s of annealing and elongation at 60 °C. Primers used are listed in Table S3. All samples were analyzed in triplicates, and primers are listed in Table S2. The relative fold ratio was calculated using ΔΔCT and log-transformed [94].

## 5. Conclusions

**Figure 11:**
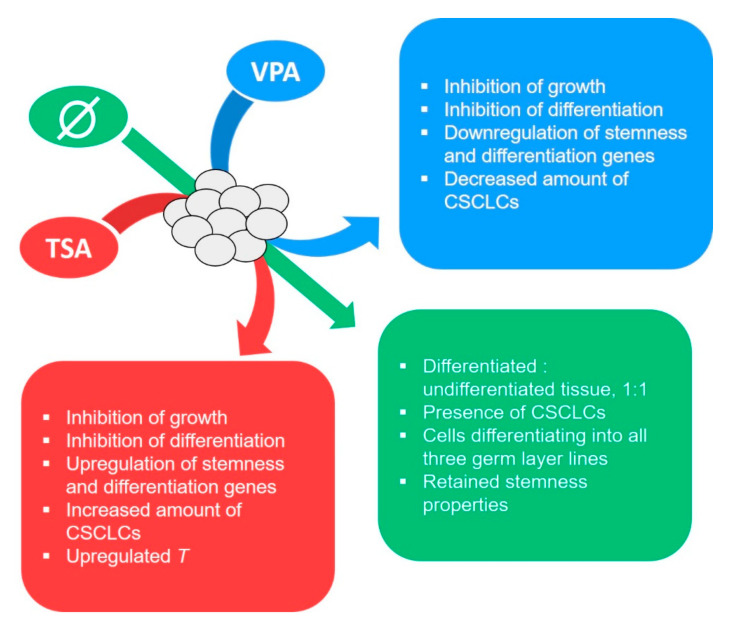
Graphical depiction of key conclusions.

The TSA and VPA treatments of the gastrulating embryo-proper have confirmed their anti-tumor effects on experimental teratomas grown in vitro, or, put differently, the inhibition of histone deacetylation by both HDACi treatments diminished the inherent tumorigenic growth potential of the normal mammalian embryonic tissue. The teratomas from TSA-treated embryos, in contrast to VPA-treated ones, had a marked increase in both CSCLC amount and a worse outcome-prognosis gene expression, which points to the possible dangers of the TSA anti-tumor treatment (Figure 11), while VPA seems to have quietened the tumor core altogether. This difference between VPA’s and TSA’s effects could be either the result of the difference in mechanisms of HDAC inhibition, with TSA being a total class I and II inhibitor and VPA not inhibiting IIb, due to differences in acetylation levels, or even some other mechanism. At the same time, this research gave a new insight into the epigenetic mechanism important for the induction of teratoma growth from the normal embryonic tissue.

## Supplementary Materials

The following are available online at https://www.mdpi.com/2072-6694/12/11/3416/s1, Table S1: Antibodies used in IHC, Table S2: Antibodies used in Western blot, Table S3: Primer sequences used in qPCR, Figure S1: Western blots with densitometry data.
