# Supplementary material for: Teratoma Growth Retardation by HDACi Treatment of the Tumor Embryonal Source
Source: Cancers (Basel). 2020 Nov 18;12(11):3416. doi: 10.3390/cancers12113416 (PMC7698704; doi:10.3390/cancers12113416)
Supplement: Supplementary file 1 [file cancers-12-03416-s001.pdf]

# Supplementary Materials: Teratoma Growth Retardation by HDACi Treatment of the Tumor Embryonal Source

**Jure Krasic, Lucija Skara, Monika Ulamec, Ana Katusic Bojanac, Sanja Dabelic, Floriana Bulic-Jakus, Davor Jezek and Nino Sincic**

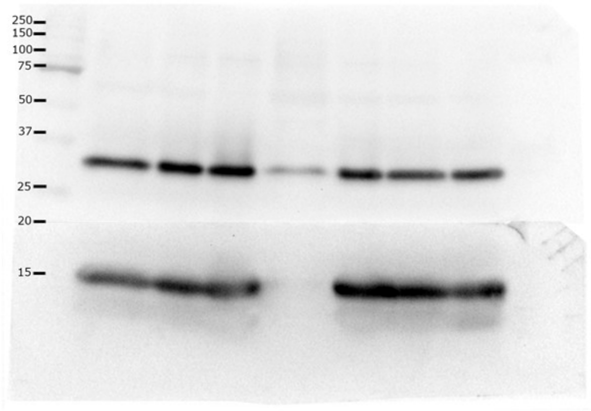


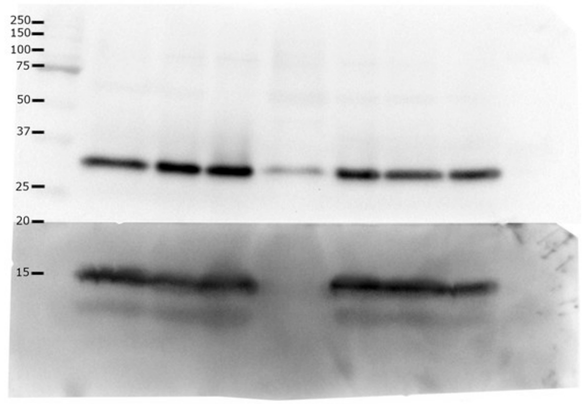


| Histone H2B - sc-8650 | | | | | | | |
|---|---|---|---|---|---|---|---|
| Lane No. | Group | Adj. Total Band Vol. (Int) | Total Band Vol. (Int) | Adj. Total Lane Vol. (Int) | Total Lane Vol. (Int) | Bkgd. Vol. (Int) | Norm. Factor |
| 1 | Other | 10094472 | 18596772 | 10606678 | 41519518 | 30912840 | 1,49 |
| 2 | Other | 8732880 | 15659244 | 9142470 | 32180436 | 23037966 | 1,72 |
| **3** | **Trichostatin A** | **10809760** | **19731205** | **11418810** | **40866475** | **29447665** | **1,39** |
| 4 | N/A | N/A | N/A | N/A | N/A | N/A | N/A |
| **5** | **Control** | **15012450** | **26211300** | **15650325** | **51067275** | **35416950** | **1,00** |
| **6** | **Valproate** | **9563076** | **17900784** | **10092438** | **36420246** | **26327808** | **1,57** |
| 7 | Other | 10145376 | 18532584 | 11199096 | 39989376 | 28790280 | 1,48 |

| Histone H3 acetyl K9 - ab10812 | | | | | | | | |
|---|---|---|---|---|---|---|---|---|
| Lane No. | Group | Adj. Total Band Vol. (Int) | Total Band Vol. (Int) | Adj. Total Lane Vol. (Int) | Total Lane Vol. (Int) | Bkgd. Vol. (Int) | Norm. Factor | Norm. Vol. (Int) |
| 1 | Other | 14468625 | 38791200 | 18049200 | 133863525 | 115814325 | 1,49 | 21558251 |
| 2 | Other | 6913395 | 25312590 | 9728235 | 92500830 | 82772595 | 1,72 | 11891039 |
| **3** | **Trichostatin A** | **12051975** | **48556560** | **17138485** | **154150165** | **137011680** | **1,39** | **16752245** |
| 4 | N/A | N/A | N/A | N/A | N/A | N/A | N/A | N/A |
| **5** | **Control** | **11124690** | **33219326** | **14298856** | **124571356** | **110272500** | **1,00** | **11124690** |
| **6** | **Valproate** | **12325383** | **35798112** | **16756110** | **131134437** | **114378327** | **1,57** | **19350851** |
| 7 | Other | 6436044 | 21629754 | 15643962 | 96683706 | 81039744 | 1,48 | 9525345 |

**Figure S1.** Western blots with densitometry data.

**Table S1.** Antibodies used in IHC.

| Antibody | Catalog Number | Manufacturer | Dilution | HIER | Secondary | Catalog Number | Manufacturer | Dilution |
|---|---|---|---|---|---|---|---|---|
| Anti-Oct4 | NBP2-67748 | Novus Biologicus | 1:150 | pH9 Tris-EDTA | Goat anti-rabbit IgG | ab97051 | Abcam | 1:1000 |
| Anti-Nanog | ab106465 | Abcam | 1:200 | pH9 Tris-EDTA | Goat anti-rabbit IgG | ab97051 | Abcam | 1:1000 |
| Anti-p53 | sc-126 | Santa Cruz Biotechnology | 1:500 | pH6 citrate buffer | REAL EnVision Detection System | K500711 | Dako | ready to use |
| Anti-Ki-67 | ab15580 | Abcam | 1:500 | pH6 citrate buffer | Goat anti-rabbit IgG | ab97051 | Abcam | 1:1000 |
| Anti-Caspase-3 | ab13847 | Abcam | 1:50 | pH6 citrate buffer | Goat anti-rabbit IgG | ab97051 | Abcam | 1:1000 |
| Anti-8-OHdG | sc-66036 | Santa Cruz Biotechnology | 1:500 | pH6 citrate buffer | REAL EnVision Detection System | K500711 | Dako | ready to use |

**Table S2.** Antibodies used in Western blot.

| Antibody. | Catalog Number | Manufacturer | Dilution | Secondary | Catalog Number | Manufacturer | Dilution |
|---|---|---|---|---|---|---|---|
| Anti-Histone H3 (acetyl K9) | ab10812 | Abcam | 1:500 | Goat anti-rabbit IgG | ab97051 | Abcam | 1:10,000 |
| Anti-Histone H2B | sc-8650 | Santa Cruz Biotechnology | 1:200 | Donkey anti-goat IgG | sc-2033 | Santa Cruz Biotechnology | 1:20,000 |

**Table S3.** Primer sequences used in qPCR.

| Gene | | Sequence | Annealing/Extension Temperature |
|---|---|---|---|
| *Pou5f1* | F | 5′-AGT TGG CGT GGA GAC TTT GC-3′ | 60 °C |
| | R | 5′-CAG GGC TTT CAT GTC CTG G-3′ | |
| *Nanog* | F | 5′-TTG CTT ACA AGG GTC TGC TAC T-3′ | 60 °C |
| | R | 5′-ACT GGT AGA AGA ATC AGG GCT-3′ | |
| *Sox2* | F | 5′-GCG GAG TGG AAA CTT TTG TCC-3′ | 60 °C |
| | R | 5′-CGG GAA GCG TGT ACT TAT CCT T-3′ | |
| *Eomes* | F | 5′-CCT GGT GGT GTT TTG TTG TG-3′ | 60 °C |
| | R | 5′-TTT AAT AGC ACC GGG CAC TC-3′ | |
| *Nestin* | F | 5′-CCC TGA AGT CGA GGA GCT G-3′ | 60 °C |
| | R | 5′-CTG CTG CAC CTC TAA GCG A-3′ | |
| *Six3* | F | 5′-CCG GAA GAG TTG TCC ATG TTC-3′ | 60 °C |
| | R | 5′-CGA CTC GTG TTT GTT GAT GGC-3′ | |
| *Fgf5* | F | 5′-GCT GTG TCT CAG GGG ATT GT-3′ | 60 °C |
| | R | 5′-CAC TCT CGG CCT GTC TTT TC-3′ | |
| *Myod* | F | 5′-CCA CTC CGG GAC ATA GAC TTG-3′ | 60 °C |
| | R | 5′-AAA AGC GCA GGT CTG GTG AG-3′ | |
| *Brachyury* | F | 5′-CTC GGA TTC ACA TCG TGA GAG-3′ | 60 °C |
| | R | 5′-AAG GCT TTA GCA AAT GGG TTG TA-3′ | |
| *Nodal* | F | 5′-CCT GGA GCG CAT TTG GAT G-3′ | 60 °C |
| | R | 5′-ACT TTT CTG CTC GAC TGG ACA-3′ | |
| *Gata4* | F | 5′-CCC TAC CCA GCC TAC ATG G-3′ | 60 °C |
| | R | 5′-ACA TAT CGA GAT TGG GGT GTC T-3′ | |
| *Sox17* | F | 5′-CGA GCC AAA GCG GAG TCT C-3′ | 60 °C |
| | R | 5′-TGC CAA GGT CAA CGC CTT C-3′ | |
| *Cer1* | F | 5′-CTC TGG GGA AGG CAG ACC TAT-3′ | 60 °C |
| | R | 5′-CCA CAA ACA GAT CCG GCT T-3′ | |
| *Rplp0* | F | 5′-TCC AGG CTT TGG GCA TCA-3′ | 60 °C |
| | R | 5′-CTT TAT CAG CTG CAC ATC ACT CAG A-3′ | |

© 2020 by the authors. Licensee MDPI, Basel, Switzerland. This article is an open access article distributed under the terms and conditions of the Creative Commons Attribution (CC BY) license (http://creativecommons.org/licenses/by/4.0/).
